# Timing of Acupuncture during LTP-Like Plasticity Induced by Paired-Associative Stimulation

**DOI:** 10.1155/2019/9278270

**Published:** 2019-04-04

**Authors:** Xiao-kuo He, Qian-qian Sun, Hui-hua Liu, Xin-yi Guo, Cheng Chen, Li-dian Chen

**Affiliations:** ^1^Fujian University of Traditional Chinese Medicine, Fuzhou, Fujian Province, China; ^2^Taihe Hospital (Affiliated Hospital of Hubei University of Medicine), Shiyan, Hubei Province, China; ^3^School of Rehabilitation Medicine, Fujian University of Traditional Chinese Medicine, Fuzhou, Fujian Province, China; ^4^Sun Yat-Sen Memorial Hospital, Sun Yat-Sen University, Guangzhou, Guangdong Province, China

## Abstract

The aim of this study was to investigate the time-dependent effects of acupuncture on the excitability and long-term potentiation- (LTP-) like plasticity induced by paired-associative stimulation (PAS) over the primary motor cortex (M1). The present examination is the first to report the influence of acupuncture on the motor-evoked potential (MEP) throughout the treatment process, including baseline (before acupuncture), the needle in situ, and the needle removal. Subsequently, the LTP-like plasticity induced by paired-associative stimulation (PAS) was explored, which consisted of 200 pairs of electrical stimulation of the ulnar nerve at the first dorsal interosseous (FDI), followed by transcranial magnetic stimulation (TMS) over the bilateral M1. TMS-MEP amplitudes over the bilateral M1 in resting conditions were measured throughout the whole treatment process. Finally, we confirmed the behavioral measurements. Significant changes were found in both the contralateral and ipsilateral acupuncture sizes as compared to the baseline values. Our results indicated that acupuncture modulated the excitability of M1, and the synaptic plasticity was time-dependent. We concluded that acupuncture should be combined with rehabilitation techniques to improve the motor function in stroke patients. Therefore, we put forward the combined application of the acupuncture timing and rehabilitation for higher therapeutic effectiveness. This trial was registered in the Chinese Clinical Trial Registry (registration no. ChiCTR-IPR-1900020515).

## 1. Introduction

Acupuncture is an ancient Chinese therapeutic method which has rapidly gained popularity all over the world. A number of randomized controlled trials have indicated its efficacy in the treatment of neurological disorders [1, 2]. Until now, it has been applied to stroke patients with motor deficits and led to a remarkable motor recovery for decades [3]. However, its mechanism of action remains uncertain, although some studies provided further evidence in favor of the capacity of acupuncture to affect motor cortex excitability and plasticity [4–7].

The effects of acupuncture on TMS measures of the motor cortex excitability have been previously examined [4–7]. The adult human M1 maintained the capacity to activity-dependent plastic change subsequent to motor practice [8] or transcranial magnetic/electrical stimulations [9]. Paired-associative stimulation (PAS) was found to induce long-lasting bidirectional after-effects on M1 excitability depending on the interval between a pair of associative stimulation protocols [10]; these after-effects are referred to as long-term potentiation- (LTP-) like and long-term depression- (LTD-) like plasticity as they are thought to be dependent on mechanisms similar to those of synaptic plasticity studied at the cellular level [11].

Evidence suggests that the combination of acupuncture with rehabilitation training, including physical therapy (PT), occupational therapy (OT), and speech and language therapy (SLT), may be effective for the treatment of poststroke neurological impairment and dysfunction, such as motor dysfunction, dysphagia, depression, urinary incontinence, and apoplectic aphasia [12]. A new guideline for adult stroke rehabilitation and recovery strongly suggested that the initiation of rehabilitation therapy (defined as PT, OT, and SLT) is beneficial as soon as the patient was able to tolerate it [13]. Most stroke rehabilitation protocols are based on motor learning to induce neural plasticity, which refers to the ability of the brain to develop new neuronal interconnections, acquire new functions, and compensate for impairment, that is, practice plasticity [14–16].

One currently important concept of neuroplasticity is proposed in the Bienenstock-Cooper-Munro (BCM) theory [17]; the introduction of a time-variable synaptic modification threshold, below which postsynaptic responses lead to long-term depression (LTD), and above which postsynaptic responses result in LTP. In the combination of different stroke neurorehabilitation therapies, the timing of these programs is to be considered in the context of Hebbian plasticity and homeostatic metaplasticity [18].

Previous studies have suggested that acupuncture caused enduring changes in cortical excitability by influencing the activity of neural synapsis, instead of the neuronal membrane [19]. Our previous investigations indicated that the modulating functions of the interneuron, which is affected by electroacupuncture, could enhance the synaptic transmission efficiency and lead to an increase in LTP in rats [20]. However, all the above studies: first, indicated only that acupuncture modulated the motor cortex excitability in acupuncture applied in situ or by removal in a single point (or some points) of time. To the best of our knowledge, no study has dynamically explored the whole process of acupuncture, including baseline (before acupuncture), the needle in situ, and removal time points. In other words, whether the motor cortex excitability modulated by acupuncture is time-dependent, it should still be elucidated. Second, the results from these earlier studies did not indicate the effect of acupuncture on LTP-plasticity. Third, whether these changes are correlated with the beneficial effect on the behavioral improvement has not yet been clarified.

We have previously established that the application of electroacupuncture at acupoints promoted the proliferation and differentiation of endogenous neural stem cells in the middle cerebral artery occlusion and reperfusion (MCAO/R) rats via enhancing the Notch signal pathways and inhibition of neuroinflammation [21–23]. The results indicated that electroacupuncture at the acupoints, especially Quchi and Zusanli, eventually improved the recovery of the motor or cognitive functions [21–23].

Therefore, the aim of the present study was to ascertain whether the changes in the cortical excitability before, during, and after acupuncture are time-dependent. In addition, we attempted to elucidate whether the time-dependent effects of acupuncture on cortical excitability induced LTP-like plasticity, increased motor learning in the human motor cortex, and whether that effect improved the motor function. In this study, MEP amplitudes were chosen as measures of neuroplasticity changes because they are predominantly influenced by changes in the synaptic excitability, which was evidenced by alterations in the presence of pharmacological modifiers of the synaptic transmission [24].

## 2. Materials and Methods

### 2.1. Participants

Eighteen healthy volunteers (ten males and eight females; 18–34 years old, mean: 23.30 ± 4.945) with no history of a head trauma, neurological disease, or other medical problems or reported contraindication to TMS participated in the study. All participants gave written informed consent. The protocols were approved by the Ethics Committee of Taihe Hospital (Affiliated Hospital of Hubei University of Medicine) of China (approval no. 2014001-2). All protocols were performed in accordance with the Ethical Principles for Medical Research Involving Human Participants of the Declaration of Helsinki [25]. All participants were Chinese and right-handed, as determined by the Edinburgh handedness inventory. They were all naive to acupuncture stimulation.

### 2.2. Transcranial Magnetic Stimulation

Participants were seated comfortably in a reclining chair. MEPs were recorded from the FDI of each hand with the active electrode over the motor point and the reference electrode on the metacarpophalangeal joint [26]. Silver/silver chloride electrodes were used contralaterally or ipsilaterally to the acupuncture sites in bipolar configuration (interdetection spacing of approximately 2 cm). We used a figure-of-eight double coil (70 mm), with a Magstim Super Rapid magnetic stimulator (YRD CCY-1, Yiruide, Wuhan, China) in this experiment. TMS was applied to the motor cortex, contralaterally or ipsilaterally to the EMG recorded muscles. The coil was orientated at 45° oblique to the sagittal plane, so that the induced current flowed perpendicularly to the estimated alignment of the central sulcus. The scalp site at which MEPs were elicited in the FDI muscle at the lowest stimulus strength was determined. Once the optimal scalp site was found, the coil was securely fixed in place by an appropriate mechanical device. We used three laser pens to locate and marked with a soft-tipped pen to assure a constant placement of the coil throughout the session. FDI muscle was recorded in all the experiments.

The response resting motor threshold (rMT) was defined as the stimulus intensity at which 5/10 consecutive single stimuli at the optimal site evoked an MEP of at least 50 uV amplitude in the relaxed muscle [27]. Stimulus intensity during the entire stimulation paradigm was set at 120% rMT of the FDI. The rMT was measured one day before the test.

### 2.3. Paired-Associative Stimulation

Paired-associative stimulation (PAS) consisted of 200 pairs (rate, 0.1 Hz) of electrical stimulation of the left ulnar nerve at the FDI muscle followed by TMS of the M1 at the optimal site for eliciting MEPs in the left FDI muscle. Electrical stimulation was applied through a bipolar electrode (cathode proximal) using constant-current square-wave pulses (duration of 1 ms) at an intensity of three times the perceptual threshold. The intensity of TMS was set at 80% rMT of the FDI. Two interstimulus intervals between the FDI muscle and TMS were used in a session; it equaled the individual N20-latency of the ulnar nerve (FDI muscle) somatosensory-evoked cortical potential plus 5 ms. These or similar intervals produced a long-lasting LTP-like increase in MEP amplitude [28]. Because the level of attention may have considerable effects on the magnitude of a PAS effect [29], the level of attention was controlled by instructing the participants to watch the stimulated hand, to count the number of the stimulus pairs, and to report this number at the end of the PAS intervention. PAS-induced plasticity was assessed by the post-PAS/pre-PAS ratio of the peak-to-peak MEP amplitudes in the resting FDI muscle of the left hand. Complete voluntary relaxation of the FDI was monitored audiovisually using high-gain EMG (50 uV/division). Trials contaminated with voluntary EMG activity were discarded from the analysis.

PAS consisted of PAS_before_ (baseline), PAS_in_ (needle with in situ), and PAS_off_ (after the needle removal). In a pilot experiment (data were not shown), we demonstrated that TMS application to the motor cortex did not produce any overt change in the MEP amplitude.

### 2.4. Behavioral Measurements

In motor practice (MP), the participants maintained an isometric contraction of the FDI by abducting the index finger against a strain gauge attached to the proximal interphalangeal joint of the index finger repeatedly (rate: 0.1 Hz, by an auditory “go” signal).

Intervention (acupuncture) was given when the subject maintained a constant small (approximately 10% of the maximum) voluntary contraction of the FDI on the left in a twitch-like fashion [30]. TMS-evoked motor output was measured as MEP amplitude in the FDI, as described above.

### 2.5. Intervention

The participants lay comfortably in a supine position, with their head immobilized by a polystyrene-bead vacuum splint molded on the neck and the rear part of the head. Acupuncture needling was performed by experienced acupuncturists under aseptic conditions. Disposable sterilized, 0.30 × 30 mm, Hawato needles (Suzhou Medical Appliance Factory, China) were used for acupuncture. The acupoints *Quchi* (LI-11) and *Waiguan* (TB-5) at the region of the left hand were used. These are two of the most frequently exploited points in Chinese acupuncture for the treatment of poststroke motor dysfunction [31, 32]. The needling methods of “lifting and thrusting” and no “rotating” were conducted on each point until the sensation of *De Qi* (a characteristic sensation of aching and tingling) was reported by the participants. Then, the needles were kept in situ without further stimulation. Disposable, noninvasive “Streitberger” needles (Asia-med, Germany) with blunt, retractable push-back tips (diameter of 0.30 mm and length of 30 mm) served as sham needles [33].

### 2.6. Experimental Design

**Figure 1:**
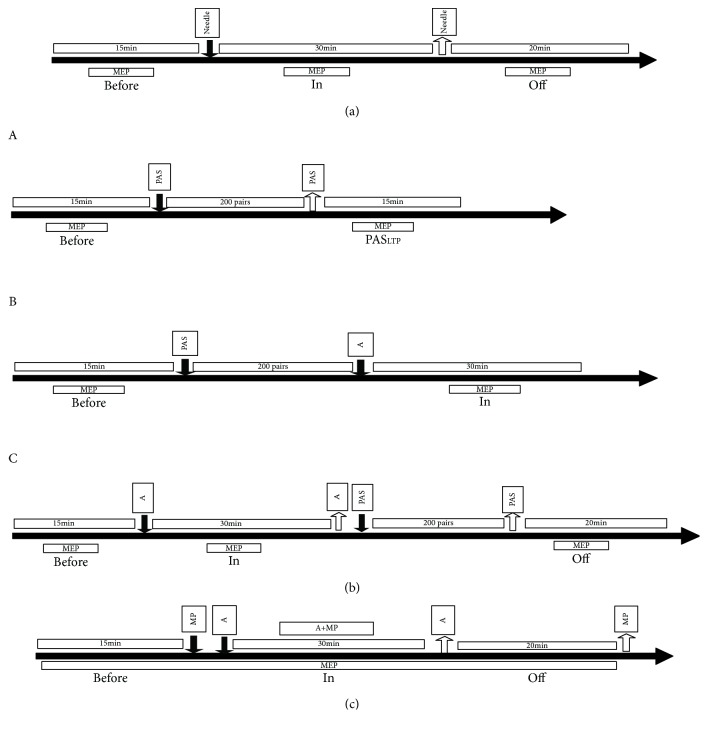
Experimental design. (a) MEP amplitudes induced by TMS over the bilateral M1 in resting conditions were measured at the “Before” (15 min before acupuncture), “In” (30 min with the needle in situ), and “Off” (30 min after the needle removal) phases, at 30-second intervals; (b) the LTP-like plasticity induced by PAS was explored, TMS-MEP amplitudes over the bilateral M1 in resting conditions were measured at the “Before” (A), “In” (B), and “Off” (C) phases; and (c) MP was tested. The average of MEP amplitudes (bilateral) were assessed at time points Before, MP (motor practice), A (acupuncture with in situ), A+MP (acupuncture+motor practice), and Off by normalization to the baseline.

The study was divided into three parts (Figure 1). In Figure 1(a), the effect of acupuncture on the motor cortex excitability was determined. Eighteen participants were randomly divided into an acupuncture group (*n* = 9) and a sham acupuncture group (*n* = 9) using the random number table method. MEP amplitudes induced by TMS over the bilateral M1 in resting conditions were measured at the “Before” (15 min before acupuncture), “In” (30 min with the needle in situ), and “After”(30 min after the needle removal) phases, at 30-second intervals (Figure 1(a)). In Figure 1(b), the LTP-like plasticity induced by PAS was explored (acupuncture group; *n* = 9). TMS-MEP amplitudes over the bilateral M1 in resting conditions were measured at the “Before,” “In,” and “After” points (Figure 1(b)). In Figure 1(c), MP was tested.

## 3. Statistical Analysis

STATA software (version 14.0, Shiyan, Taihe Hospital) was used for statistical analysis. A one-way repeated measure ANOVA was performed to compare the differences in mean MEP amplitudes over three-time phase's bilateral hemispheres (baseline, with a needle in situ, and after needle removal). PAS-induced plasticity was assessed by the post-PAS/pre-PAS ratio of the peak-to-peak MEP amplitudes in the resting FDI muscle of the left hand. *Post hoc* pairwise comparisons or one-sample two-tailed *t*-tests were performed. The Bonferroni correction was applied to adjust for multiple comparisons. The significance level was set at *P* < 0.05. Unless otherwise stated, values are reported as mean ± standard deviation.

## 4. Results

### 4.1. Needling Sensation

Participants well-tolerated the acupuncture procedures in all the experimental sessions. Needling was experienced as a short-lasting pain sensation (pricking) for all acupuncture sites. Sham needling was reported as a mild pain sensation, which was no different from acupoint needling. In traditional Chinese medicine, *De Qi* is a unique sensation of numbness, soreness, heaviness, or tingling that develops at the site of acupuncture, often spreading towards nearby cutaneous areas. When acupuncture was performed along classical meridians (both at LI-11 and TB-5 on the hand), *De Qi* sensation was reported following needle insertion in the acupoints in 90% of the experimental sessions. This sensation was not felt during sham needling.

### 4.2. Time Dependence of the Acute Effects of Acupuncture on MEP Amplitudes and Motor Cortex Excitability

Significant changes in the mean MEP amplitudes from their baseline values were observed with acupoint needling which was not seen with sham needling. The mean MEP amplitudes before needling were averaged and normalized to 1.

**Figure 2:**
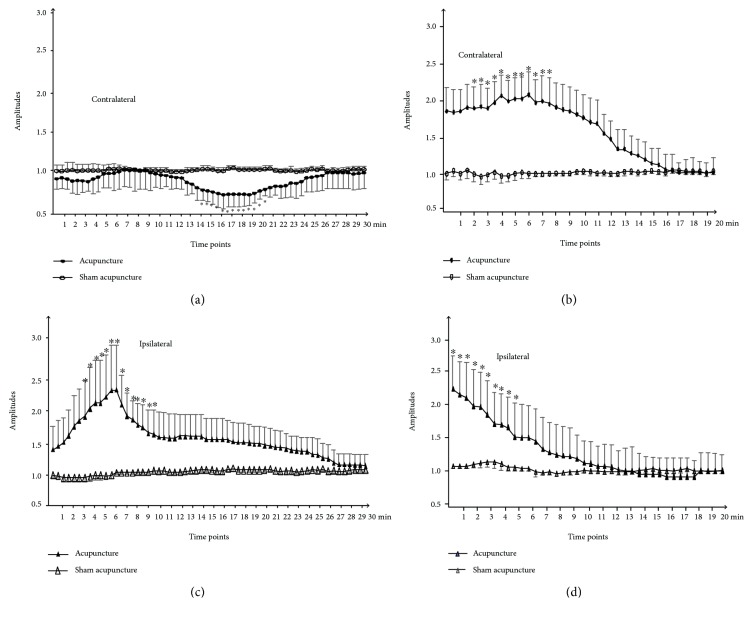
Effects of the acupuncture on MEP amplitude. (a) Amplitude changes of MEPs in FDI muscles following the needle insertion in situ on the contralateral hemisphere of the acupuncture sites. (b) Amplitude changes of MEPs in FDI muscles after the needle removal from the in situ on the contralateral hemisphere of the acupuncture sites. (c) Amplitude changes of MEPs in FDI muscles following the needle insertion in situ on the ipsilateral hemisphere of the acupuncture sites. (d) Amplitude changes of MEPs in FDI muscles following the needle insertion in situ on the ipsilateral hemisphere of the acupuncture sites.

Changes in the MEP amplitudes occurred on both the contralateral and ipsilateral sides (Figure 2). However, the direction of the change and its time course were markedly different, the latter of which outlasted the time period of needle application (Figures 2(a) and 2(c)), including the needle removal (Figures 2(b) and 2(d)), depending on the acupuncture side. No significant changes were established after the sham needling treatment on both sides (Figure 2).

Mean MEP amplitude of each point obtained in the corresponding phase of the experimental protocol. Note that the steadiness of the waveform amplitudes can readily be observed before and after needle manipulation, which is maintained also after needle removal.

The effects of acupuncture on the contralateral average MEP recordings from the FDI muscle in all participants, including the in situ needle and after its removal, can be seen in Figures 2(a) and 2(b). The 5–7 min duration resulted in slight depression of MEP amplitudes seen with the needle in situ (*P* > 0.05). Then, the cortical excitability slowly returned to its baseline values; when the needle was in situ, the excitability started to decline at approximately 12 min, which continued until the 20 min time point (*P* < 0.001), followed by a decline back to the baseline level at 25 min. After the needling removal, the MEPs were significantly facilitated from baseline to 8–10 min (*P* < 0.001), then declined to the baseline level at 15 min.

Figures 2(c) and 2(d) illustrate the effects of acupuncture on the ipsilateral average MEP recordings from FDI muscle. Acupoint needling resulted in significantly facilitated MEP amplitudes compared with the baseline when 4–10 min with the needle in situ (*P* < 0.001). The excitability of the cortical circuits began to rise when the needle was removed within five minutes (*P* < 0.001), and then started to decrease until 20 min, followed by its return to the baseline level.

**Figure 3:**
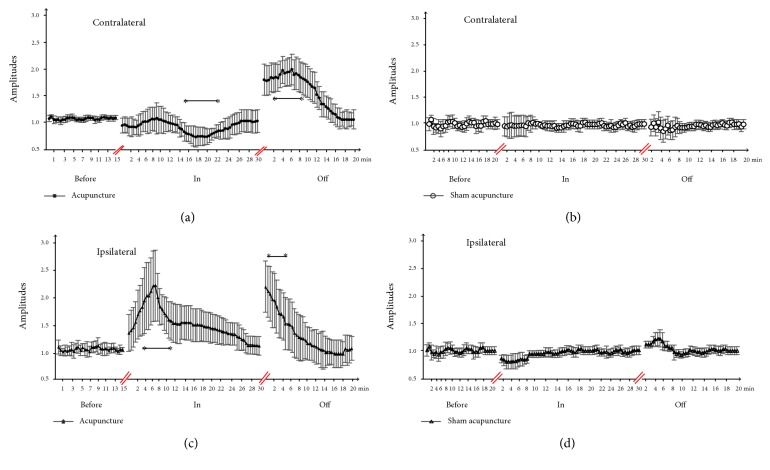
(a, c) Amplitude modulation of MEPs in the left FDI muscle following acupuncture during the different phases of the experimental protocol and (b, d) amplitude modulation of MEPs in the left FDI muscle following sham acupuncture groups during the different phases of the experimental protocol. The “Before” panel indicates the baseline before acupuncture application, whereas the “In” panel denotes the average values across participants for the experiments in which the needle was in situ for 30 minutes. The “Off” panel refers to the data recorded after the needle removal within 20 minutes. Graphical illustration of the mean MEP amplitudes over the whole acupuncture is presented. The asterisks indicate the statistical significance compared with the baseline. The levels of significance are as indicated in the text.

The mean MEP amplitudes used contralaterally to the acupuncture sites were 0.91 ± 0.142 in acupuncture and 1.22 ± 0.357 after acupuncture. In contrast, no significant changes on contralateral or ipsilateral motor cortex excitability were seen with sham needling when the needle in situ or removal after. The mean MEP amplitudes for the ipsilateral to the acupuncture sites were 1.31 ± 0.409 in acupuncture and 1.26 ± 0.279 after acupuncture. No changes were found in the sham needling groups (Figure 3).

Our findings showed that acupoint needling resulted in MEP amplitudes that were significantly lower than those at the baseline for the needle in situ (*F*_(1, 16)_ = 35.55; *P* < 0.001) and facilitated MEP amplitudes after needle removal (*F*_(1, 16)_ = 23.16; *P* < 0.001) for the contralateral cortical (the acupuncture sites). Similarly, for the ipsilateral cortical, it also showed significant effects of the needle in situ (*F*_(1, 16)_ = 14.02; *P* < 0.001) and after needle removal (*F*_(1, 16)_ = 20.88; *P* < 0.001). There was no significance in the sham needling group.

### 4.3. Time Dependence of LTP-like Plasticity on Acute Acupuncture Effects of PAS on Corticospinal Excitability

The PAS intervention, with the needle removal (*P* < 0.001), resulted in significantly facilitated MEP amplitudes after the needle removal for the contralateral of the acupuncture sizes. MEP amplitudes in the needle in situ significantly decreased. The mean MEPs were 0.98 ± 0.042 with needle in situ and 1.10 ± 0.079 after the needle removal.

**Figure 4:**
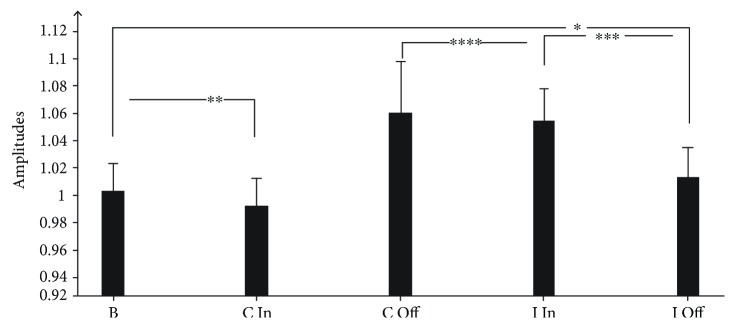
Paired-associative stimulation (PAS)-induces plasticity. PAS effects in sessions B, C In, C Off, I In, and I Off were assessed by the average of motor-evoked potential (MEP) amplitudes at time points Before (B), Contralateral Acupuncture In (C In), Contralateral Acupuncture Off (C Off), and Ipsilateral Acupuncture In (I In), Ipsilateral Acupuncture Off (I Off) normalized to the baseline. All data are mean ± SD of nine participants. The asterisks indicate the statistical significance (one-way repeated measure ANOVA).

The PAS intervention with the needle in situ and removal significantly facilitated MEP amplitudes (1.09 ± 0.049 versus 1.02 ± 0.046; *P* < 0.001, respectively) for the ipsilateral acupuncture sizes (Figure 4).

### 4.4. Results from the Behavioral Test

**Figure 5:**
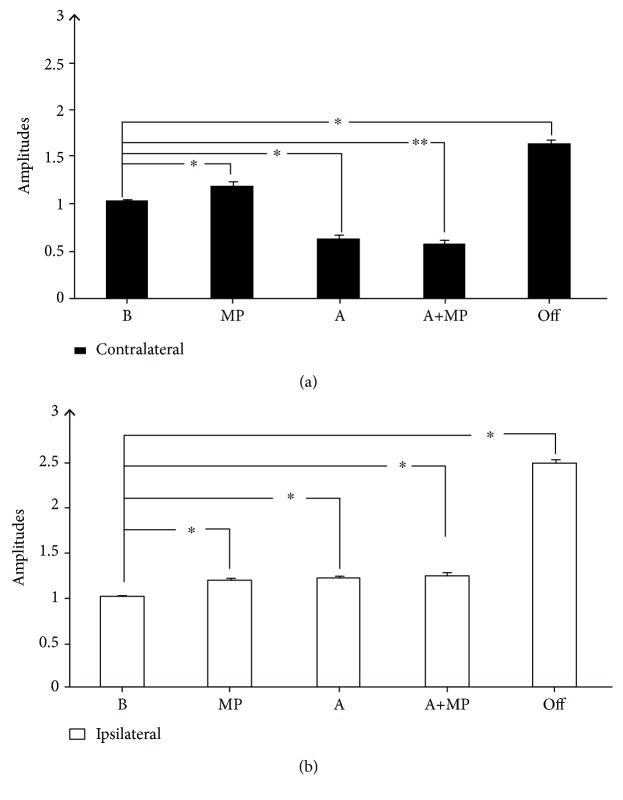
Results of the behavioral test. In B (before), MP (motor practice), A (in-situ acupuncture), A+MP (acupuncture+motor practice), and Off were assessed by the average of MEP amplitudes (a) contralateral and (b) ipsilateral at time points B, MP, A, A+MP, and Off normalized to the baseline. All data are mean ± SD of nine participants. The asterisks indicate statistical significance (one-way repeated measure ANOVA).

MP caused an increase in FDI muscle MEP amplitude as compared with the baseline values (1.16 ± 0.041; *P* < 0.001), whereas a decrease in MEP amplitude (0.61 ± 0.041; *P* < 0.001) occurred when pretreatment with acupuncture was implemented. The combined treatment with acupuncture and MP led to a slight decrease in the MEP amplitudes (0.56 ± 0.037; *P* = 0.004). The needle removal (1.58 ± 0.043; *P* < 0.001) significantly facilitated MEP amplitudes after needle removal for the contralateral of the acupuncture sizes (Figure 5(a)).

However, compared with the baseline, MP, Acupuncture, and Acupuncture+MP caused an increase in FDI muscle MEP amplitude (1.16 ± 0.041; *P* < 0.001; 1.19 ± 0.043; *P* < 0.001; 1.22 ± 0.049, *P* < 0.001, respectively). The needle removal significantly facilitated MEP amplitudes for the ipsilateral of acupuncture sizes (2.47 ± 0.054; *P* < 0.001; Figure 5(b)).

## 5. Discussion

Acupuncture, a traditional Chinese therapeutic method, has been widely used in clinical practice to treat diseases, such as stroke, Alzheimer's disease, Parkinson's disease, dysmenorrhea, and chronic pain [34]. Mounting data suggested that electroacupuncture alleviated dementia and restored the long-term potentiation in rats. Clinical evidence also indicated that electroacupuncture improved the electrical activity of the brain in vascular dementia patients. According to the BCM theory, the changes in cortical excitability affect the plasticity.

Our results demonstrated that the excitability and the LTP-like plasticity of the bilateral M1 were modulated by acupuncture with single *Quchi* and *Waiguan* used as acupoints.

Several pertinent aspects of the findings should be emphasized. First, our results showed for the first time the effects of acupuncture on MEPs throughout the entire treatment process, including before acupuncture, the needle in situ, and the needle removal. Second, the observed modulation of muscle excitability can considerably outlast 20 minutes after the needle removal, demonstrating that acupuncture was able to induce LTP-like plasticity changes in the cortical. Third, our work confirmed that the time-dependent effects of acupuncture on cortical excitability induce LTP-like plasticity and increased motor learning in the human motor cortex.

Until now, a few studies have investigated the effects of acupuncture on the TMS measures of motor system excitability. They explored the influence of MEP amplitudes when the needle was inserted in situ or after its removal at single time points. The results of these previous examinations are in accordance with our findings, which confirmed the effects of acupuncture on the contralateral average MEP recordings from FDI muscle in all participants including with the needle in situ and after its removal. The excitability started to decline at approximately 12 min which continued until the 20 min point (*P* < 0.001). After the removal of the needle, the MEPs were significantly promoted as compared to their baseline levels to 2–8 min. Furthermore, the acupoint needling resulted in the significantly facilitated MEP amplitudes comparable to those at the baseline when the needle was in situ for 4–10 min, whereas the excitability of the cortical began to rise when the needle was removed after 5 min.

Synaptic plasticity in the form of LTP and LTD is widely considered to be involved in at least some forms of motor learning. Metaplasticity is one prominent concept of activity-dependent plasticity regulation that encompasses the changes in the synaptic and/or neuronal state, which shape the direction, magnitude, and duration of future synaptic changes [35]. The basic idea of the metaplasticity theory is that the threshold for activity-dependent synaptic plasticity is not static but dynamic.

The currently most influential theory of metaplasticity is the BCM theory of bidirectional synaptic plasticity [36]. The crucial assumption is not constant but varies as a function of previous activity of the postsynaptic neuron. The theory postulates the existence of a modification of sliding threshold for synaptic plasticity.

Recent evidence shows that the extent of practice-dependent plasticity in the motor cortex can be purposefully enhanced by experimental manipulation. One way of improving motor learning is to transiently increase the excitability of the motor cortex during motor learning [37, 38]. This can be achieved by weakening concurrently the excitability of the intracortical inhibitory circuits with practice. This principle is being referred to as “gating.” The theory is that a given stimulation or learning protocol may not result in any overt change in synaptic strength if applied under “normal” postsynaptic excitability conditions. In contrast, if applied at a time when the integrated postsynaptic response of the stimulated cortex is enhanced by disinhibiting or depolarizing experimental manipulation, such as temporary ischemic nerve block or excitability-enhancing transcranial brain stimulation, then the same stimulation or learning protocol leads to an increase in the synaptic strength [37].

Another strategy to boost learning is to decrease the threshold for the induction of synaptic plasticity by lowering the neuronal activity in the motor cortex before practice. This approach invokes homeostatic metaplasticity.

In the present investigation, we found that the contralateral mean MEP amplitudes significantly were lower than baseline levels after PAS when in situ acupuncture was applied, whereas the mean MEP amplitudes increased significantly after needle removal. Additionally, the ipsilateral mean MEP amplitudes were augmented when the needle was inserted and removed; we implemented PAS at these time points and found that the MEP amplitudes were higher than before. These findings might be linked to “gating.” One important mechanism of gating is the removal of a voltage-sensitive magnesium block from NMDA receptors during depolarization, which enhances the intracellular calcium entry and hence the postsynaptic response by the same stimulation or learning protocol.

Our findings suggest that LTP-like plasticity can be manipulated to modulate the plasticity of the human cortex by acupuncture at different time points, such as those used in our protocol. This leads to the notion that practice-dependent cortical plasticity and the associated changes in behavior, such as perceptual and motor learning and the recovery of functions after the occurrence of lesions, can be promoted when concomitant measures are taken to induce LTP-like plasticity mechanisms in the cortex.

Despite the recent progress in rehabilitation techniques, the recovery of the motor function after stroke is usually difficult and incomplete. However, certain techniques can be used to induce the central nervous system to produce “practice-dependent plasticity” by enhancing the sensory and motor performance to the affected hemisphere, such as PT/OT/ST. These rehabilitation techniques have been suggested to play an important role in the process of motor recovery [39, 40]. However, more than 60% of the stroke survivors suffer from persistent neurological deficits and impaired dexterity, which causes a significant negative impact on their daily living activities (dressing, eating, and self-care) and independence. Thus, development of new strategies that promote stroke recovery to be used in addition to the classical rehabilitation methods is highly required [41].

Our findings indicate that the bilateral human primary motor cortex excitability and the LTP-like plasticity can be modulated by acupuncture at the *Quchi* (LI-11) and *Waiguan* (TB-5) acupoints, but the effect in healthy volunteers depended on the timing. Our results confirmed that the increase in the TMS-evoked motor cortical output was not merely an epiphenomenon but is associated with a parallel improvement in the behavioral measures.

## 6. Conclusion

The results of the present study indicate that acupuncture can modulate the excitability of M1 and the plasticity is time-dependent.

We suggested that the combination of acupuncture and rehabilitation techniques substantially attenuated the motor function impairment after stroke. Moreover, the initiation of rehabilitation therapy (defined as PT, OT, and SLT) as soon as the needle was removed exerted even more beneficial effects.

## Data Availability

All data included in this study are available upon request by contact with the corresponding author.
